# Notes to the Taxonomic Affiliation of the *Bulbophyllym* Sect. *Physometra* (Orchidaceae, Epidendroideae) Based on Molecular Phylogenetic Analyses

**DOI:** 10.3390/ijms24119709

**Published:** 2023-06-03

**Authors:** Sławomir Nowak, Natalia Olędrzyńska, Dariusz L. Szlachetko, Magdalena Dudek

**Affiliations:** Department of Plant Taxonomy and Nature Conservation, Faculty of Biology, The University of Gdansk, Wita Stwosza 59, 80-308 Gdansk, Poland; slawomir.nowak@ug.edu.pl (S.N.);

**Keywords:** *Bulbophyllum physometrum*, ITS, low copy gene, Orchidaceae, phylogeny, plastid marker

## Abstract

To solve the taxonomic affiliation of *Bulbophyllum physometrum*, the only known species of the *Bulbophyllym* sect. *Physometra* (Orchidaceae, Epidendroideae), we conducted phylogenetic analyses based on nuclear markers, i.e., ITS and the low-copy gene *Xdh*, and the plastid region *mat*K. We used Asian *Bulbophyllum* taxa, with a special focus on species from the sections *Lemniscata* and *Blepharistes*, i.e., the only Asian sections of this genus with bifoliate pseudobulbs, as in *B*. *physometrum*. Unexpectedly, the results of molecular phylogenetic analyses showed that *B*. *physometrum* is most probably more related to the representatives of the sections *Hirtula* and *Sestochilos* than *Blepharistes* or *Lemniscata*.

## 1. Introduction

*Bulbophyllum* Thouars is the largest pantropical genus of Orchidaceae, including more than 2000 species [1], most of which are restricted to rainforest habitats [2,3]. The richest area in species of this genus and at the same time the greatest morphological diversity we can observe in the Paleotropical region. There are hundreds of *Bulbophyllum* species in Central and Southeast Asia [2,4,5]. Often, some of them are known only from a single locality and are characterized by a set of unique features that makes it difficult to classify them into any of the proposed sections. Phylogenetic relationships within such a diverse genus also remain unresolved. Studies indicate that taxa occurring outside Asia represent separate evolutionary lineages, and their evolution in the Neotropics [6] or Madagascar [7] has been more extensively examined. In the case of Asian taxa, there are works covering comprehensively selected groups, e.g., the section *Cirrhopetalum* [8], but due to the number of species found in Asia, representation in phylogenetic studies of the entire genus is still poor. The results obtained, however, indicate that many sections are non-monophyletic [3].

**Figure 1:**
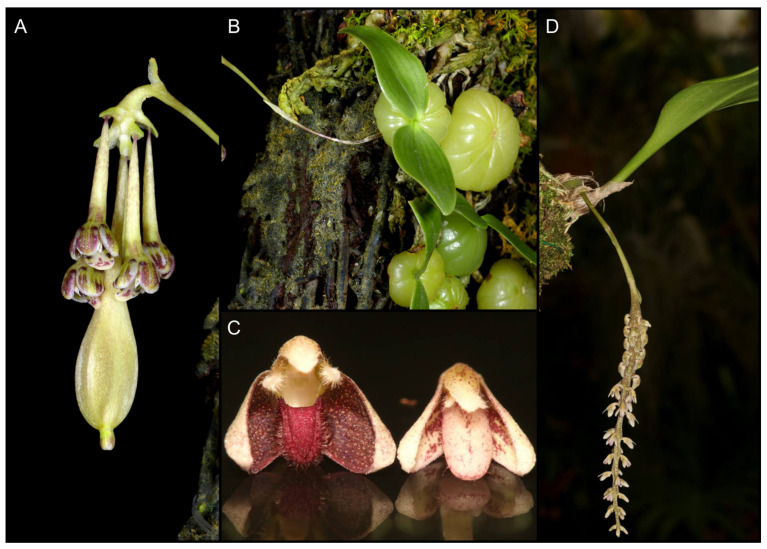
Two *Bulbophyllum* species with dimorphic flowers. Inflorescence with two types of flowers (**A**) and two-leaved pseudobulb (**B**) of *B. physometrum,* photos by Ron Parsons. Fertile (on the left) and sterile (on the right) flowers (**C**), and inflorescence (**D**) of *B. mirabile,* photos by Roland Amsler.

One of the species whose phylogenetic position is unknown is *B*. *physometrum*, described by Vermeulen and his collaborators [9]. It is an epiphytic taxon that has been found in the northern part of Thailand (Mae Hong Son Province) and is known in only two localities. Therefore, the authors propose to consider it as endangered [9]. Moreover, in this unique species, we can observe dimorphism in the morphology of its flowers. It consists in the fact that the apical flower in the inflorescence is sterile, and its floral segments are much smaller than in fertile ones. In addition, its ovary is large and remarkably inflated [9]. Floral dimorphism is a rare phenomenon in *Bulbophyllum*. The only other species possessing this feature is *B*. *mirabile* Hallier [10], although in the latter species, differences between flowers are not so prominent as in *B. physometrum* (Figure 1). Another unique feature of *B*. *physometrum* is two-leaved pseudobulbs, much more common in African and Madagascan species than in Asian ones. Within the Asiatic *Bulbophyllum* bifoliate pseudobulbs, it can only be found in two sections: *Lemniscata* Pfitzer and *Blepharistes* J.J.Verm., Schuit. & De Vogel [9]. Despite some morphological similarities, the authors of *B*. *physometrum* did not classify it into any of the mentioned sections. Based on vegetative and floral structures, they proposed a new monotypic section *Physometra* J.J.Vermeulen, Suksathan & Watthana.

In our study, we conducted molecular phylogenetic analyses to determine the relationship of *B*. *physometrum* to other Asian representatives of the genus. For this purpose, we used two nuclear markers (ITS and *Xdh*), and the plastid gene *mat*K. To date, one of these markers (ITS) has been successfully used in the phylogeny of both African (including Malagasy) and Neotropical representatives of *Bulbophyllum* [6,7]. Further, Hu et al. [8] performed molecular phylogenetics of the *Cirrhopetalum* alliance based on mentioned markers. *Bulbophyllum physometrum* was sampled twice. In addition, we also used samples representing species from the sections *Lemniscata*, *Blepharistes*, and *Hirtula* in our analyses. Unfortunately, we were unable to obtain a sample for *B*. *mirabile* Hallier.

## 2. Results

**Figure 2:**
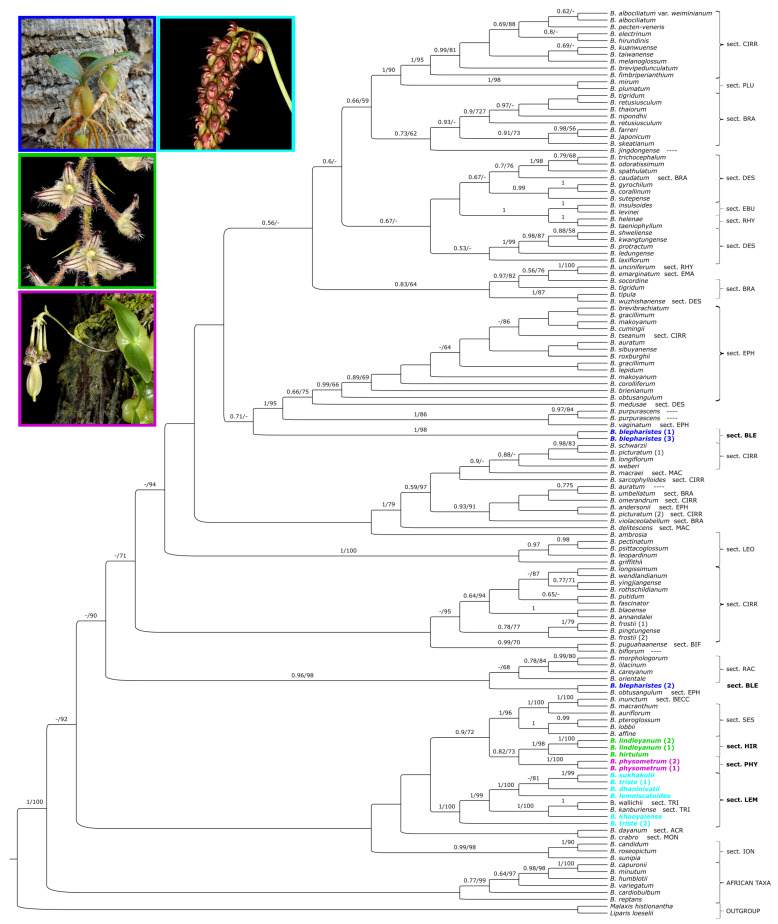
The maximum clade credibility tree obtained for nuclear combined dataset (ITS−*Xdh*) showing the phylogenetic position of the Asian monotypic section *Physometra*. The numbers above branches indicate values of posterior probability and bootstrap support (PP/BS). Sectional placement for particular studied taxa is shown behind the taxon name or bracketed together as abbreviations of three or four letters: ACR−*Acrochaene*, BECC−*Beccariana*, BIF−*Biflorae*, BRA−*Brachyantha*, BLE−*Blepharistes*, CIRR−*Cirrhopetalum*, DES−*Desmosanthes*, EBU−*Eublepharon*, EMA−*Emarginatae*, EPH−*Ephippium*, HIR−*Hirtula*, ION−*Ione*, LEM−*Lemniscata*, LEO−*Leopardinae*, MAC−*Macrostylida*, MON−*Monomeria*, PHY−*Physometra*, PLU−*Plumata*, RAC-*Racemosae*, RHY−*Rhytionanthos*, SES−*Sestochilos*, TRI−*Tripudianthes*. However, lack of section placement is denoted as ----. The sections discussed are highlighted in a color other than black. Photos of key species representing the sections *B. blepharistes* (photo by Sławomir Nowak), *B. lindleyanum, B. physometrum*, and *B. triste* (photos by Ron Parsons) are marked with a box in the same color.

**Figure 3:**
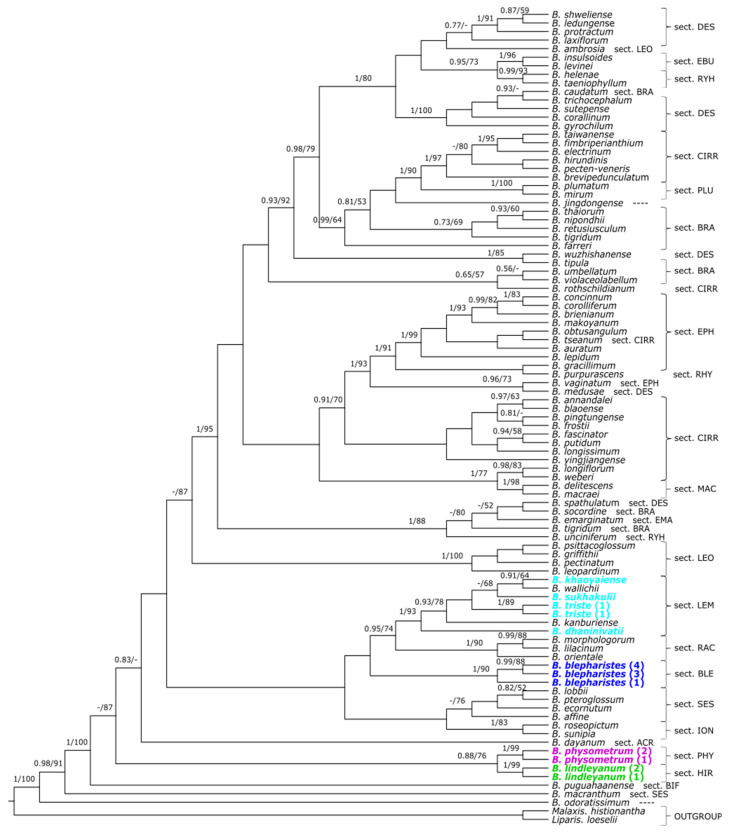
The maximum clade credibility tree obtained for dataset of *mat*K gene showing the phylogenetic position of the Asian monotypic section *Physometra*. The numbers above branches indicate values of posterior probability and bootstrap support (PP/BS). Sectional placement for particular studied taxa is shown behind the taxon name or bracketed together as abbreviations of three or four letters: ACR−*Acrochaene*, BECC−*Beccariana*, BIF−*Biflorae*, BRA−*Brachyantha*, BLE−*Blepharistes*, CIRR−*Cirrhopetalum*, DES−*Desmosanthes*, EBU−*Eublepharon*, EMA−*Emarginatae*, EPH−*Ephippium*, HIR−*Hirtula*, ION−*Ione*, LEM−*Lemniscata*, LEO−*Leopardinae*, MAC−*Macrostylida*, MON−*Monomeria*, PHY−*Physometra*, PLU−*Plumata*, RAC−*Racemosae*, RHY−*Rhytionanthos*, SES-*Sestochilos*, TRI−*Tripudianthes*. However, lack of section placement is denoted as ----. The sections discussed are highlighted in a color other than black.

**Figure 4:**
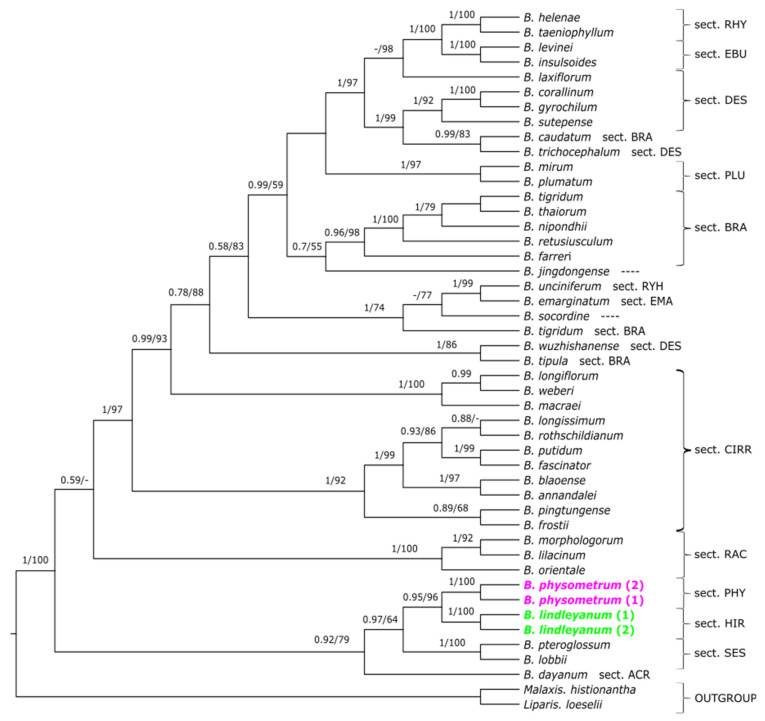
The maximum clade credibility tree obtained for combined dataset (ITS−*Xdh*−*mat*K) showing the phylogenetic position of the Asian monotypic section *Physometra*. The numbers above branches indicate values of posterior probability and bootstrap support (PP/BS). Sectional placement for particular studied taxa is shown behind the taxon name or bracketed together as abbreviations of three or four letters: ACR−*Acrochaene*, BECC−*Beccariana*, BIF−*Biflorae*, BRA−*Brachyantha*, BLE−*Blepharistes*, CIRR−*Cirrhopetalum*, DES−*Desmosanthes*, EBU−*Eublepharon*, EMA−*Emarginatae*, EPH−*Ephippium*, HIR−*Hirtula*, ION−*Ione*, LEM−*Lemniscata*, LEO−*Leopardinae*, MAC−*Macrostylida*, MON−*Monomeria*, PHY−*Physometra*, PLU−*Plumata*, RAC−*Racemosae*, RHY−*Rhytionanthos*, SES−*Sestochilos*. However, lack of section placement is denoted as ----.The sections discussed are highlighted in a color other than black.

For the dataset of single markers, a similar tree topology was obtained in both used methods. Therefore, we present maximum clade credibility trees (Figure 2, Figure 3 and Figure 4) with the BI analysis, but bootstrap support (BS) with the ML analysis was also placed. On the trees obtained for the combined nuclear matrix (ITS-*Xdh*) (Figure 2) and plastid *mat*K gene (Figure 3), *B. physometrum* (sampled twice) are grouped with the representatives of section *Hirtula*. However, this clade has not received strong support on the node (PP = 0.82/BS = 73; PP = 0.88/BS = 76). Additionally, in the case of the tree for the combined nuclear matrix, a clade including taxa from sections *Sestochilos* and *B*. *inunctum* (sect. *Beccariana*) is sister to it. We observe a somewhat different situation on the cladogram obtained for the combined matrix for all markers (ITS-*Xdh*-*mat*K) (Figure 4). On this tree, *B*. *physometrum* is joined together with *B*. *lindleyanum* (sect. *Hirtula*) into a clade strongly supported on the node, but only by the posterior probability value (PP = 0.95), and moderately supported by the bootstrap value (BS = 76), whereas species of the section *Sestochilos* (*B*. *lobii* and *B*. *pteroglossum*) are sister to them. Representatives of the section *Lemniscata* formed a monophyletic group on both the ITS-*Xdh* and *mat*K trees; however, this is a clade whose evolutionary line evolved independently of *B*. *physometrum*. Similarly, a clade representing *B*. *blepharistes* (quadruple sampled in this study) (sect. *Blepharistes*) on the *mat*K tree formed an independent line from the group of taxa where *B*. *physometrum* was placed. On the other hand, on the ITS-*Xdh* tree, one *B*. *blepharistes* sample labeled as 2 joined together with species of the section *Racemosae*. However, the other two (*B*. *blepharistes* 1 and *B*. *blepharistes* 3) have similarly formed a distinct line from *B*. *physometrum* on the *mat*K tree.

## 3. Discussion

### 3.1. Phylogeny of Bulbophyllum

A comprehensive phylogenetic study is still lacking for the genus *Bulbophyllum*. Undoubtedly, a technical limitation is the enormous species diversity of this largest orchid genus. Many species have never been sampled in molecular studies, and only a few groups have been relatively extensively studied, such as the Neotropical clade [6], the Malagasy clade [7], or the sect. *Cirrhopetalum sensu lato* [8]. Most studies succeed in obtaining a clade resolution at the section level, but the relationships between clades are usually poorly supported or absent [6,7,8,11]. Such a pattern can also be observed in our results (Figure 2, Figure 3 and Figure 4), so the phylogenetic relationships within the entire genus are difficult to interpret, but it is still possible to indicate the species’ sectional affiliation in most cases. Another problem is the fact of monophyly of the sampled infrageneric groups. With limited sampling, many sections seemingly can appear monophyletic, but with increasing taxon sampling, it seems that the lack of monophyly is widely observed [8] (Figure 2). Many sections, based on a limited range of morphological characters, represent high species diversity, such as the sect. *Cirrhopetalum* and the sect. *Hirtula*, so this result can be expected in these groups. Our results also confirm the paraphyletic nature of sections, such as *Brachyantha*, *Desmosanthes*, and *Cirrhopetalum*, for example (Figure 4). Such a result is also often due to the position of some taxa within the group, e.g., *B. wallichii* and *B. kanburiense* from the sect. *Tripudianthes* inside the sect. *Lemniscata* (Figure 2). However, it should be noted that due to less extensive sampling, i.e., paraphyletism of the sect. *Hirtula* is not observed, although other research results indicate it [11]. Another important aspect is the correctness of the labeled samples, which can be an important issue with a genus as large as *Bulbophyllum*. The position of *B. blepharistes 2* on the tree based on nuclear markers (Figure 2) is most likely due to a mislabeled sample.

The selection of a molecular marker remains another important element in the study of *Bulbophyllum* phylogeny. The studies show that the use of traditional markers that provide a solution in other orchid groups does not provide a solution here [6,7,8,11], although it seems that nuclear markers are much more informative than plastid markers [6,8,12]. The reason is probably the evolutionary history of the group, especially the oldest clade of Asian taxa, at the same time the most diverse [12]. Promising results are provided by the *Xdh* marker used in the analyses of the evolution of the *Cirrhopetalum* group [8] and this study only. At the same time, the *matK* marker does not resolve many relationships, by low or no support for them, likewise many other plastid markers. In further approaches to reconstructing phylogeny, it is worth testing other areas of the plastome that are indicated as more informative, so being specific hotspots for the genus [13,14]. Of course, phylogenomic analyses can provide valuable results, but they seem to be challenging with such a large group. 

In summary, the results of the phylogeny of the genus indicate that a revision of the infrageneric classification of *Bulbophyllum* is highly recommended for the genus. 

### 3.2. Relationship with the Section Hirtula

The results obtained for phylogenetic analyses with moderate support (PP = 0.82, 0.88, 0.95; BS = 73, 76) indicate that *B. physometrum* is related to representatives of the section *Hirtula* (Figure 2, Figure 3 and Figure 4). However, the section *Hirtula* includes 35–45 species, depending on the author, and poses a taxonomic problem in itself. Until now, one phylogenetic work on this group of species has appeared [11]. Unfortunately it embraced only *B*. *lindleyanum*, *B*. *limbatum*, *B*. *hirtulum*, and *B*. *dayanum*. The GeneBank resources contain sequences of some markers, mainly ITS, of only a few species of the section. Analyses involving the aforementioned species indicate the paraphyletic nature of the section [11], but the study of the section’s phylogeny should be regarded as very preliminary.

**Figure 5:**
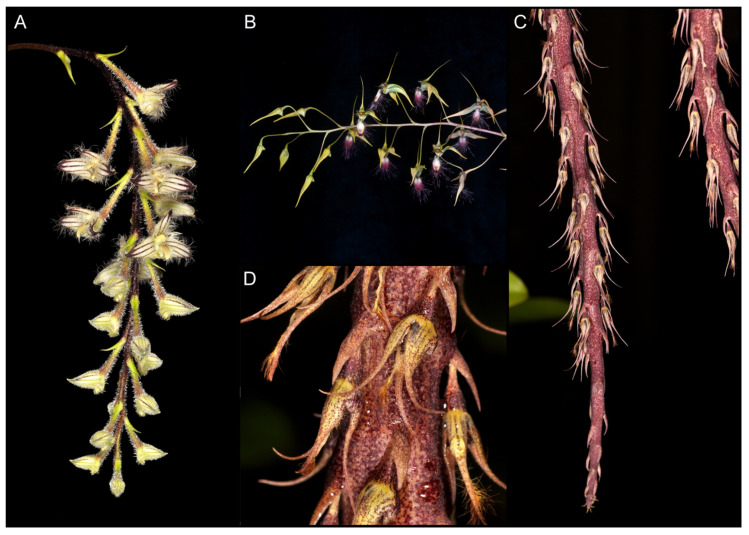
Inflorescence variation in *Bulbophyllum* sect. *Hirtula*: *B. lindleyanum* (**A**), *B. jolandae* (**B**), and *B. clipeibulbum* (**C**,**D**). Photos by Ron Parsons.

The *Hirtula* group is fairly diverse in terms of flowers morphology and inflorescence organization. In fact, it is very difficult to list features unique to this section or present in all *Hirtula* species, except for single-leafed pseudobulbs, but this one is widely common, especially in Asiatic representatives of the genus. However, one can easily divide the section *Hirtula* into three groups based on the flowers’ arrangements: species with cylindrical, more or less swollen inflorescence axis; species with thin, wiry inflorescence; and species with a more or less subumbellate inflorescence (Figure 5).

In the flowers arrangements, *B. physometrum* seems to be similar to the 3rd of the mentioned group. A rather unique feature of *B. physometrum* is the exceptionally long pedicel, far exceeding the length of ovary and floral segments. This is different in *B. mirabile*, where the subsessile ovary is equal or subequal to the perianth segments. In the section *Hirtula*, it is easy to find a group of species in which we observe situations similar to that in *B. physometrum*, i.e., slender pedicel much longer than the sepals—*B. jolandae, B. lasioglossum, B. lindleyanum, B. nigripetalum, B. secundum,* and *B. tremulum* (Figure 5).

### 3.3. Flowers Dimorphism

Flower dimorphism can have various backgrounds. One of the most common is sexual dimorphism when male and female flowers differ significantly in structure, size, or even floral scent [15,16,17,18], which is related to their different functions and participation in different stages of pollination [18,19,20]. Such a phenomenon can also be observed among orchids, although it is very rare. Flowers with sexual dimorphism have species of the genus *Catasetum* Rich. ex Kunth [21,22], both in terms of their structure and the scent they emit, which is an important aspect of their pollination biology.

The second example of dimorphic flowers, where they are often all fertile and bisexual, is the significant differences in their size [23,24]. This is most often related to the fact that the flowers are gathered in inflorescences and have different sizes in their individual parts [23,25,26]. Such a phenomenon has also been well documented in representatives of the Orchidaceae, with examples within European orchids [27].

Finally, an example of flower dimorphism is the presence of sterile flowers. They are often highly transformed, so that they are significantly different from those that perform reproductive functions. In addition to the various functions that sterile flowers can serve, such as male flowers in trap inflorescences in Araceae [28,29], studies indicate that the presence of sterile flowers paradoxically increases pollinator visits and promotes reproductive success [30,31,32]. Within the orchids, such a phenomenon can also be observed. An example is the species of the Neotropical genus *Heteranthocidium* Szlach., Mytnik & Romowicz. In representatives of this taxon within a single inflorescence, some of the flowers are stellate and sterile [33]. Another example is *Bulbophyllum physometrum*. However, here, only one terminal flower in the inflorescence is sterile; moreover, the major difference is due to the significant enlargement of the ovary, so that it looks like a seed capsule [9]. Although this phenomenon is rare in orchids, there is another species of the genus *Bulbophyllum* exhibiting sterile flowers syndrome: *B. mirabile* (section *Hirtula*) [34]. In this case, however, the flowers vary in the size and structure of the perianth, such as the lip, depending on their position on the inflorescence. Flowers located at the top are usually sterile, while those in the basal part are normally developed (Figure 1). Phylogenetic analyses indicate that *B. physometrum* is related to representatives of the section *Hirtula,* i.e., *B. lindleyanum* and *B. hirtula* (Figure 2). However, the previously indicated differences in flower dimorphism, as well as inflorescence structure, may suggest independent evolution of the flower dimorphism phenomenon in both species. The rachis of *B. physometrum* is spindle-shape, shortened, and somewhat swollen at the apex, while *B. mirabile* has an elongated and cylindrical rachis, clearly swollen near the middle. Finally, *B. physometrum* has a two-leaved pseudobulb, while representatives of the section *Hirtula* are characterized by a one-leaf pseudobulb [9,34].

### 3.4. Two-Leaved Pseudobulb

**Figure 6:**
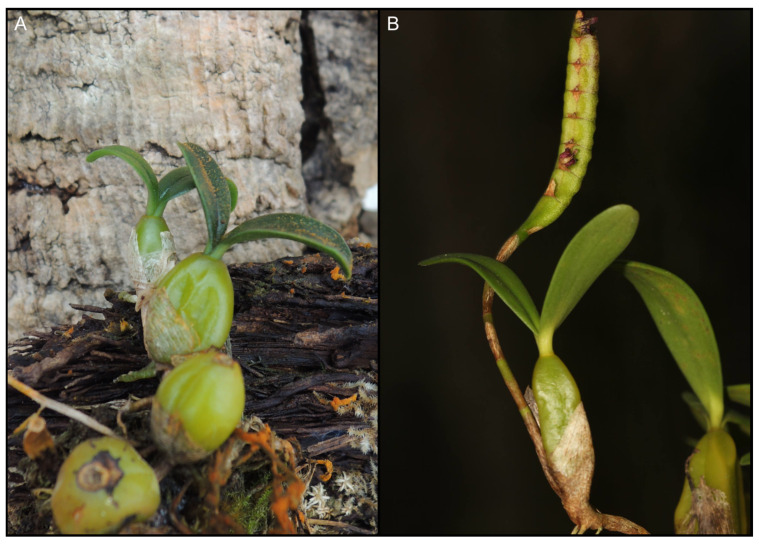
*Bulbophyllum* species with two-leaved pseudobulb: *B. blepharistes* (**A**) and African *B.* cf. *scaberulum* (**B**). Photos by Sławomir Nowak (**A**) and Roland Amsler (**B**).

Another feature that *B. physometrum* has, and which is also rare in Asian representatives of the genus, is a pseudobulb with two leaves. Phylogenetic studies have indicated that *B. physometrum* is unlikely to be closely related to other representatives with such a trait, i.e., species belonging to the sect. *Blepharistes* (Figure 6) and the sect. *Lemniscata* (Figure 2 and Figure 3). Both in the tree obtained for the nuclear dataset, i.e., ITS-*Xdh* (Figure 1), and for the plastid gene *mat*K (Figure 2), each of the mentioned sections is an independent evolutionary lineage. The pseudobulb with two leaves appears much more frequently in *Bulbophyllum* species found in Africa [35], Madagascar [7], and the Neotropics [6,36] (Figure 6). Therefore, this feature undoubtedly evolved independently in *Bulbophyllum* representatives.

### 3.5. Mimicry of Flower or Fruit?

One of the most striking features of *B. physometrum* is the presence of a sterile flower, but at the same time, its ovary is strongly enlarged. It is hard not to get the impression that with the often complicated pollination systems in orchids, such a transformed flower may play an important role in attracting pollinators and may be an element of mimicry.

*Bulbophyllum physometrum* is found in lowland forest, a floristically rich vegetation type in northern Thailand. The transformed flower can resemble the flowers of other co-occurring plant groups, and thus, *B. physometrum* can benefit from their pollinator pool. This phenomenon is known in other groups of orchids [37,38,39]. The shape of the sterile flower may resemble some flowers that the Aristolochiaceae have, or plant families that have inferior ovaries, as in the Cucurbitaceae. Another possibility is that it resembles the entire sympetalous flower, such as in many Ericaceae. All the families mentioned are rich in species, but they also certainly do not cover all the possibilities of structures that the sterile flower of *B. physometrum* could resemble.

Alternatively, with the greatly reduced perianth elements, it is possible that such a structure is deceptively similar to a fruit. Therefore, one may wonder whether *B. physometrum* is not the first case among orchids where we have a mimicry of a fruit-like flower. Such a phenomenon is known in the plant world, although it is relatively rare [40]. It should be remembered here that the mimicry of a flower is related to the higher success of its pollination. Therefore, a flower resembling the fruit would attract pollinators to properly develop flowers. Orchid seeds are largely dispersed by the wind. Very rarely, animals are attracted by the orchid’s fruit [41,42]. Fruits can produce several secondary metabolites [43,44], but it is not known whether they can be significant in attracting pollinators. Certainly, however, plant metabolites play a very important role in attracting pollinators [45]. Pollination by fruit flies has been confirmed in various *Bulbophyllum* species, but the greatest role is played by chemical compounds secreted by the flower [46,47,48]. Further research is needed to confirm whether it is possible that the fruit-like flower of *B. physometrum* may be a mimicry-based strategy to promote pollination. A valuable source of information may be the determination of chemical compounds secreted by both types of *B. physometrum* flowers.

## 4. Materials and Methods

For phylogenetic reconstruction, we sampled taxa of *Bulbophyllum* that represent the Asiatic sections of this genus, and *Liparis loeselii* (L.) Rich. and *Malaxis histionantha* (Otto) Garay & Dunst. as an outgroup were chosen based on the results of Givinsh et al. [49]. All the sequences obtained and downloaded with GenBank (www.ncbi.nlm.nih.gov) are presented in the supplementary material (Table S1). The leaf fragments used in the molecular analyses were taken from plants cultivated in the greenhouse of the University of Gdansk and the Botanical Garden of Heidelberg, and a private collection from Tadeusz Kusibab. The vouchers of samples that we obtained new sequences of are presented in Table 1.

DNA Isolation. Total genomic DNA was extracted from 20 mg of dried leaves [50] using the DNA Sherlock AX Kit (A&A Biotechnology, Gdańsk, Poland), following the manufacturer’s protocol. The homogenization of samples was performed by a FastPrep instrument (MP Biomedicals, Santa Ana, CA, USA). Pellets of DNA were suspended in 50 µL of TE buffer. The quantity and purity of the isolated DNA were determined and checked using NanoDrop One of Thermo Scientific.

Amplification and sequencing. The PCRs for the markers (ITS, *Xdh*, *mat*K) were performed in a total volume of 25 µL containing 1 µL temple DNA (~10–100 ng), 0.5 µL of 10 µM of each primer, 11 µL MyTaq HS DNA Polymerase Mix (BIOLINE Ltd., London, UK), and water. The amplification parameters for nrITS (ITS1 + 5.8S + ITS2) were 94 °C, 4 min; 30× (94 °C, 45 s; 52 °C, 45 s; 72 °C, 1 min); and 72 °C, 7 min, and for the plastid marker (part of the *mat*K gene) were 95 °C, 3 min; 33× (94 °C, 45 s; 52 °C, 45 s, 72 °C, 90 s); and 72 °C, 7 min., whereas a touchdown protocol was used for PCR amplification for the low-copy gene *Xdh*. The initial denaturation step (95 °C, 5 min) was followed by 7 cycles of 94 °C, 45 s; 59 °C, 45 s (reducing 1 °C per cycle); 72 °C, 90 s. The next step involved 30× (94 °C, 45 s; 52 °C, 45 s; 72 °C, 90 s) and 72 °C, 7 min. The products of the PCR reaction were tested using electrophoresis in 1% agarose gel with a voltage of 110 V for 25 min. The Clean-Up Concentrator Kit (A&A Biotechnology, Gdańsk, Poland) was used to clean PCR products, following the manufacturer’s protocol, eluted with 30 µL of nuclease-free water. Purified PCR products were sequenced by Macrogen (Seoul, South Korea–http://dna.macrogen.com/eng/). DNA sequence chromatograms were examined/edited in FinchTV (https://finchtv.software.informer.com/1.4/).

The amplification and sequencing reactions were performed using the same pairs of primers for each marker. For the ITS region (ITS1-5.8S-ITS2), 101F and 102R primers were used [51], for the low-copy gene, *Xdh* primers X551F and X1591R [52], whereas, for the part of the *mat*K gene, primers 19F [53] and 1326R [54] were used.

Phylogenetic analyses. The DNA sequences were aligned using Mafft v.7 [55]. In the beginning, two datasets were prepared. The first (1147 bp, 132 taxa) consisted of combined nuclear markers (ITS and *Xdh*), and the second (1137 bp, 95 taxa) included only a plastid marker (*mat*K). Finally, after removing the conflicted taxa, nuclear and plastid markers were combined into one dataset (47 taxa, 2535 bp.). Information on features of the aligned datasets is presented in Table 2. The best fitting models were calculated on the PhyML website (http://www.atgc-montpellier.fr), using the AIC (Akaike Information Criterion). The GTR + I + G (ITS, *mat*K) and HKY + I + G (*Xdh*) models were chosen as the best fitting ones.

The phylogeny of the studied group was reconstructed using two different methods. The maximum likelihood analysis (ML) was conducted with 1000 bootstrap replicates in raxmlGUI 2.0 [56]. The Bayesian inference reconstruction (BI) was performed in 2 separate 4-Markov-chain Monte Carlo (MCMC) runs, each with 20,000,000 generations, in MrBayes 3.2.7a [57] on a CIPRES Science Gateway [58]. Each time, the value of the average standard deviation of split ranges was <0.01.

The results of BI analyses, the maximum clade credibility trees, were generated, with a burn-in of 25%, in TreeAnnotator v. 1.8.4. [59]. Obtained trees were edited with FigTree v.1.4.4 (http://tree.bio.ed.ac.uk/software/figtree/) and Inkscape (https://inkscape.org/release/inkscape-1.0.2/). The nodal confidence was assessed by both posterior probability values (PP) and bootstrap support (BS). In both cases, the values lower than 0.50 (PP) or 50 (BS) were removed from the trees, while values equal to or higher than 0.95 (PP) and higher than 85 (BS) were considered strong support [60,61].

## 5. Conclusions

*Bulbophyllum physometrum* shows morphological features not observed in other representatives of the genus. Hence, it is classified in a separate section, *Physometra*. Considering the differences in the overall morphological structure of the inflorescences and flowers, it can be speculated that they evolved independently. The second important feature is pseudobulbs with two leaves, but phylogenetic studies indicate a distant relationship with other species with the same feature. Again, we can presume that this trait arose independently in the process of evolution. In conclusion, based on the morphology and very preliminary results of molecular results, it seems reasonable to maintain a separate section for *B. physometrum,* as proposed by Vermeulen et al. [9], in order to emphasize the distinctiveness and uniqueness of the species. However, the relationships among many sections of the genus are still unresolved or unknown and, therefore, require expanded research with high sampling and the use of methods other than the molecular markers standard in orchid phylogeny. Further research is needed to refine the classification of Asian species in particular.

## Figures and Tables

**Table 1:** The list of plants used for the molecular study, with a place of origin and accession numbers. Numbers (1)–(3) indicate the number of the sample used for testing.

Sample Name	Place of Acquisition	Voucher Number
*Bulbophyllum blepharistes* (3)	Greenhouse of the University of Gdansk	UGDA.0000254
*Bulbophyllum lindleyanum* (1)	Botanical Garden of Heidelberg	UGDA.0073190
*Bulbophyllum physometrum* (1)	Greenhouse of the University of Gdansk	UGDA.0000001
*Bulbophyllum physometrum* (2)	Private collection of Tadeusz Kusibab (Cracow)	UGDA.0073171

**Table 2:** Information on features of the aligned datasets used in phylogenetic analyses.

Dataset	No. of Taxa	Informative Features	Uninformative Features	Constant Features	Total Features
nuclear combined	132	529	232	686	1147
*mat*K gene	95	155	109	873	1137
Nuclear + plastid (ITS1-5.8S-ITS2, *Xdh*, *mat*K)	47	462	255	1818	2535

## Data Availability

All DNA sequences obtained by the corresponding author have been deposited in the NCBI database (https://www.ncbi.nlm.nih.gov/genbank/nih.gov), and they will be made public as soon as the manuscript is accepted for publication (the DNA sequences have the following accession numbers at GenBank—ITS markers: OQ506144–OQ506147; Xdh: OQ680572-OQ680573; *mat*K: OQ680568-OQ680571). However, datasets are available from the corresponding author.

## Supplementary Materials

The following supporting information can be downloaded at: https://www.mdpi.com/article/10.3390/ijms24119709/s1.
