# Supplementary material for: Notes to the Taxonomic Affiliation of the Bulbophyllym Sect. Physometra (Orchidaceae, Epidendroideae) Based on Molecular Phylogenetic Analyses
Source: Int J Mol Sci. 2023 Jun 3;24(11):9709. doi: 10.3390/ijms24119709 (PMC10253564; doi:10.3390/ijms24119709)
Supplement: Supplementary file 1 [file ijms-24-09709-s001.zip › ijms-2333206-supplementary.pdf]

Table S1: List of taxa used in phylogenetic analyses, along with GenBank accession numbers and their distribution. Taxon names for the newly obtained sequences are highlighted in bold and blue.

Taxon	Distribution	GB no for ITS	GB no for Xdh	GB no for maK
Bulbophyllum acuminatum	Asia - tropical	MK164424.1	MK178113.1	JF305802.1
Bulbophyllum affine	Asia - temperate and tropical	EF195916.1	MK178114.1	MK178010.1
Bulbophyllum albociliatum	Asia - temperate	MK164426.1	MK178115.1	MK178011.1
Bulbophyllum albociliatum var. weiminianum	Asia - temperate	MK164531.1	MK178218.1	MK178109.1
Bulbophyllum ambrosia	Asia - temperate and tropical	MK164427.1	MK178116.1	MT518277.1
Bulbophyllum andersonii	Asia - temperate and tropical	JN619417.1	MN619014.1	KF361642.1
Bulbophyllum annandalei	Asia - temperate and tropical	KY966427.1	MK178118.1	MK178014.1
Bulbophyllum auratum	Asia - temperate and tropical	MK164431.1	MK178119.1	MK177915.1
Bulbophyllum auriflorum	Africa and Madagascar	EF195963.1	GU004448.1	MN618636.1
Bulbophyllum biflorum	Asia - tropical	MK164434.1	MK178081.1	MK178018.1
Bulbophyllum blaoense	Asia - tropical	MK164436.1	MK178123.1	MK178020.1
Bulbophyllum blepharistes (1)	Asia - tropical	MK164437.1	MK178124.1	MK178021.1
Bulbophyllum blepharistes (2)	Asia - tropical	EU477512.1	----	----
**Bulbophyllum blepharistes (3)**	Asia - tropical	OQ506144	----	OQ680568.1
Bulbophyllum blepharistes (4)	Asia - tropical	----	----	MT518280.1
Bulbophyllum brevibrachiatum	Asia – tropical	MK164438.1	MK178125.1	----
Bulbophyllum brevipedunculatum	Asia - temperate	MK164439.1	MK178126.1	MK178022.1
Bulbophyllum brienianum	Asia - tropical	MK164440.1	MK178127.1	MK178023.1
Bulbophyllum candidum	Asia - temperate and tropical	OM112090.1	KT885014.1	KT884992.1
Bulbophyllum capuronii	Africa and Madagascar	KJ558736.1	MN619022.2	----
Bulbophyllum crabro	Asia - temperate and tropical	MK164445.1	MK178132.1	----
Bulbophyllum cardiobulbum	Africa and Madagascar	EF195967.1	MN619023.1	----
Bulbophyllum careyanum	Asia - temperate and tropical	KY966435.1	MK178024.1	MW862206.1
Bulbophyllum caudatum	Asia - temperate and tropical	MK164442.1	MK178129.1	MK178025.1
Bulbophyllum corallinum	Asia - temperate and tropical	MK164443.1	MK178130.1	MK178026.1
Bulbophyllum corolliferum	Asia - temperate and tropical	MG253855.1	MK178131.1	MK178027.1

Bulbophyllum cumingii	Asia - temperate and tropical	EF195923.1	MK178133.1	----
Bulbophyllum dayanum	Asia - temperate and tropical	MK164447.1	MK178134.1	MK178028.1
Bulbophyllum delitescens	Asia - temperate and tropical	KY966442.1	MK178135.1	MK178029.1
Bulbophyllum dhaninivatii	Asia - temperate and tropical	MK164449.1	MK178136.1	MK178030.1
Bulbophyllum ecornutum	Asia - temperate and tropical	LC487577	----	KJ462091.1
Bulbophyllum electrinum	----	MK164450.1	MK178137.1	MK178031.1
Bulbophyllum emarginatum	Asia - temperate and tropical	MK164451.1	MK178138.1	MK178032.1
Bulbophyllum farreri	Asia - temperate and tropical	MK164452.1	MK178139.1	MK178033.1
Bulbophyllum fascinator	Asia - temperate and tropical	MK164453.1	MK178140.1	MK178034.1
Bulbophyllum frostii (1)	Asia - temperate and tropical	MK164456.1	MK178142.1	MK177939.1
Bulbophyllum frostii (2)	Asia - temperate and tropical	KY966445.1	----	KY966736.1
Bulbophyllum gracillimum (1)	Asia, Australasia and Pacific	MK164457.1	MK178143.1	MK178038.1
Bulbophyllum gracillimum (2)	Asia, Australasia and Pacific	KY966446.1	----	----
Bulbophyllum griffithii	Asia - temperate and tropical	MK164458.1	MK178144.1	MK178039.1
Bulbophyllum gyrochilum	Asia - temperate and tropical	MK164459.1	MK178145.1	MK178040.1
Bulbophyllum helenae	Asia - temperate and tropical	MK164460.1	MK178146.1	MK178041.1
Bulbophyllum hirtulum	Asia - temperate and tropical	JF428126.1	----	---
Bulbophyllum hirundinis	Asia - temperate and tropical	MK164461.1	MK178147.1	MK178042
Bulbophyllum humblotii	Africa and Madagascar	KJ558711.1	MN619047.1	----
Bulbophyllum insulsoides	Asia temperate	MK164462.1	MK178148.1	MK178043.1
Bulbophyllum inunctum	----	JF428110.1	KC709966.1	----
Bulbophyllum japonicum	Asia temperate	AB786894.1	MK178149.1	MK178044.1
Bulbophyllum jingdongense	----	MK164463.1	MK178150.1	MK178045.1
Bulbophyllum kanburiense	Asia - temperate and tropical	MK164464.1	MK178151.1	MK178046.1
Bulbophyllum khaoyaiense	Asia - temperate and tropical	MK164465.1	MK178152.1	MK178047.1
Bulbophyllum kuanwuense	Asia - temperate and tropical	----	MK178153.1	MK178048.1
Bulbophyllum kwangtungense	Asia - temperate	JN619414.1	MK178154.1	----
Bulbophyllum laxiflorum	Asia - temperate and tropical	KY966449.1	MK178155.1	MK178050.1
Bulbophyllum ledungense	Asia - temperate	MK164468.1	MK178156.1	MK178051.1
Bulbophyllum lemniscatoides	Asia - temperate and tropical	KY966450.1	----	----

Bulbophyllum leopardinum	Asia - temperate and tropical	MK164469.1	MK178157.1	MK178052.1
Bulbophyllum lepidum	Asia - temperate and tropical	KY966452.1	MK178158.1	MK178053.1
Bulbophyllum levinei	----	KY966453.1	MK178159.1	MK178054.1
Bulbophyllum lilacinum	Asia - temperate and tropical	JF428111.1	MK178160.1	MK178055.1
**Bulbophyllum lindleyanum (1)**	Asia - temperate and tropical	OQ506145	OQ680572	OQ680569
Bulbophyllum lindleyanum (2)	Asia - temperate and tropical	KY966454.1	----	KY966746.1
Bulbophyllum lobbii	Asia - temperate and tropical	KY966457.1	MK178161.1	KY966748.1
Bulbophyllum longiflorum	Africa, Madagascar, Asia, Pacific ans Australasia	EF196024.1	MK178162.1	MK178057.1
Bulbophyllum longissimum	Asia - temperate and tropical	KY966458.1	MK178163.1	----
Bulbophyllum macraei	Asia - temperate and tropical	MK164475.1	MK178164.1	MK178059.
Bulbophyllum macranthum	Asia - temperate and tropical	MG253850.1	KC709958.1	KJ462084.1
Bulbophyllum makoyanum (1)	Asia - temperate and tropical	MK164476.1	MK178165.1	MK178060.1
Bulbophyllum makoyanum (2)	Asia - temperate and tropical	MG253857.1	----	----
Bulbophyllum medusae	Asia - temperate and tropical	MK164477.1	MK178166.1	MK178061.1
Bulbophyllum melanoglossum	Asia - temperate	MK164478.1	MK178167.1	----
Bulbophyllum minutum	Africa and Madagaskar	KJ558732.1	MN619071.1	----
Bulbophyllum mirum	Asia - temperate and tropical	MK164479.1	MK178168.1	MK178062.1
Bulbophyllum morphologorum	Asia - temperate and tropical	MG253858.1	MK178169.1	MK178063.1
Bulbophyllum nipondhii	Asia - temperate and tropical	MK164481.1	MK178170.1	MK178064.1
Bulbophyllum obtusangulum (1)	----	JN619410.1	MK178171.1	MK178065.1
Bulbophyllum obtusangulum (2)	----	MK164482.1	----	----
Bulbophyllum odoratissimum	Asia - temperate and tropical	HQ114230.1	MK178172.1	FJ94042.1
Bulbophyllum omerandrum	Asia - temperate	JN619419.1	MK178173.1	----
Bulbophyllum orientale	Asia - temperate and tropical	MK164485.1	MK178174.1	KY966760.1
Bulbophyllum pecten-veneris	Asia - tropical	MK164486.1	MK178175.1	KY966761.1
Bulbophyllum pectinatum	Asia - temperate and tropical	MK164487.1	MK178176.1	MK178069.1
**Bulbophyllum physometrum (1)**	Asia - temperate and tropical	OQ506146	----	OQ680570
**Bulbophyllum physometrum (2)**	Asia - temperate and tropical	OQ506147	OQ680573	OQ680571
Bulbophyllum picturatum (1)	Asia - temperate and tropical	MK164488.1	MK178177.1	----
Bulbophyllum picturatum (2)	Asia - temperate and tropical	EF195939.1	MK178177.1	----

Bulbophyllum pingtungense	Asia - temperate	MK164489.1	MK178178.1	MK178070.1
Bulbophyllum plumatum	Asia - temperate and tropical	MK164490.1	MK178179.1	MK178071.1
Bulbophyllum protractum	Asia - temperate and tropical	MK164491.1	MK178180.1	MK178072.1
Bulbophyllum psittacoglossum	Asia - temperate and tropical	MK164492.1	MK178181.1	MK178073.1
Bulbophyllum pteroglossum	Asia - temperate and tropical	MK164493.1	MK178182.1	MK178074.1
Bulbophyllum puguahaanense	Asia - temperate and tropical	MK164495.1	MK178183.1	MK178075.1
Bulbophyllum purpurascens (1)	Asia - temperate and tropical	KY966473.1	MK178184.1	MK178076.1
Bulbophyllum purpurascens (2)	Asia - temperate and tropical	MK164496.1	----	----
Bulbophyllum putidum	Asia - temperate and tropical	MK164497.1	MK178185.1	MK178077.1
Bulbophyllum retusiusculum (1)	Asia - tropical	MK164500.1	MK178188.1	MK178079.1
Bulbophyllum retusiusculum (2)	Asia - tropical	MK164499.1	MK178187.1	MK178080.1
Bulbophyllum roseopictum	Asia - temperate and tropical	MK164501.1	MK178189.1	MK178081.1
Bulbophyllum rothschildianum	Asia - temperate and tropical	MK164502.1	MK178190.1	MK.178082.1
Bulbophyllum roxburghii	Asia - temperate and tropical	MK164503.1	MK178191.1	----
Bulbophyllum sarcophylloides	Asia - temperate and tropical	MK164505.1	MK178192.1	MK178084.1
Bulbophyllum schwarzii	Asia - temperate and tropical	MK164506.1	MK178193.1	----
Bulbophyllum shweliense	Asia - temperate and tropical	MK164507.1	MK178194.1	MK178085.1
Bulbophyllum sibuyanense	Asia - temperate and tropical	MK164508.1	MK178195.1	----
Bulbophyllum skeatianum	Asia - tropical	----	MK178196.1	----
Bulbophyllum socordine	Asia - temperate and tropical	MK164509.1	MK178197.1	MK178087.1
Bulbophyllum spathulatum	Asia - temperate and tropical	MK165478.1	MK178200.1	MK178090.1
Bulbophyllum sukhakulii	Asia - temperate and tropical	MK164514.1	MK178201.	MK178092.1
Bulbophyllum sunipia	Asia - temperate and tropical	MK165463.1	MK178202.1	MK178093.1
Bulbophyllum sutepense	Asia - temperate and tropical	KY966482.1	MK178203.1	MK178094.1
Bulbophyllum taeniophyllum	Asia - temperate and tropical	MK164517.1	MK178204.1	MK178095.1
Bulbophyllum taiwanense	Asia - temperate	KY022460.1	MK178205.1	MK178096.1
Bulbophyllum thaiorum	Asia - temperate and tropical	MK164519.1	MK178206.1	MK178097.1
Bulbophyllum tigridum (1)	Asia - temperate and tropical	KX455820.1	MK178207.1	MK178089.1
Bulbophyllum tigridum (2)	Asia - temperate	MK164520.1	MK178207.1	----
Bulbophyllum tipula	Asia - temperate and tropical	MK164521.1	MK178208.1	MK178099.1

Bulbophyllum trichocephalum	----	MK164522.1	MK178209.1	MK178100.1
Bulbophyllum triste (1)	Asia - temperate and tropical	MK164523.1	MK178210.1	MK178101.1
Bulbophyllum triste (2)	Asia - temperate and tropical	KY966483.1	MK178210.1	KY966775.1
Bulbophyllum tseanum	Asia - temperate	MK164524.1	MK178211.1	MK178102.1
Bulbophyllum umbellatum	Asia - temperate and tropical	KY966484.1	MK178212.1	MK178212.1
Bulbophyllum unciniferum	Asia - temperate and tropical	MK164526.1	MK178213.1	MK178104.1
Bulbophyllum vaginatum	Asia - tropical	MK164527.1	MK178214.1	MK178105.1
Bulbophyllum variegatum	Africa/Madagaskar	KJ558713.1	----	----
Bulbophyllum violaceolabellum	Asia - tropical	MK164528.1	MK178215.1	MK178106.1
Bulbophyllum wallichii	Asia - tropical	JN619411.1	MK178216.1	MK178107.1
Bulbophyllum weberi	Asia - tropical	MK164530.1	MK178217.1	MK178108.1
Bulbophyllum wendlandianum	Asia - tropical	MK164532.1	MK178219.1	----
Bulbophyllum wuzhishanense	Asia - temperate	MK164533.1	MK178220.1	MK178111.1
Bulbophyllum yingjiangense	Asia - temperate	MK164534.1	MK178221.1	MK178112.1
Liparis loeselii	----	EF079387.1	GU004450.1	KT987363.1
Malaxis histionantha	----	HG970124.1	HG970146.1	GU004449.1
